# Neuromedin B promotes chondrocyte differentiation of mesenchymal stromal cells via calcineurin and calcium signaling

**DOI:** 10.1186/s13578-021-00695-1

**Published:** 2021-10-18

**Authors:** Marie Maumus, Guillaume Fonteneau, Maxime Ruiz, Said Assou, Hassan Boukhaddaoui, Philippe Pastoureau, Frédéric De Ceuninck, Christian Jorgensen, Danièle Noel

**Affiliations:** 1 IRMB, Univ Montpellier, INSERM, Montpellier, France; 2 INM, University of Montpellier, INSERM, CHU Montpellier, Montpellier, France; 3 Institut de Recherches Servier, Center for Therapeutic Innovation, Immuno-Inflammatory Disease, Croissy-sur-Seine, France; 4 Clinical Immunology and Osteoarticular Disease Therapeutic Unit, Department of Rheumatology, CHU Montpellier, Montpellier, France

**Keywords:** Mesenchymal stromal cell, Chondrogenic differentiation, Chondrogenesis, Cartilage, Transcriptomic, NMB

## Abstract

**Background:**

Articular cartilage is a complex tissue with poor healing capacities. Current approaches for cartilage repair based on mesenchymal stromal cells (MSCs) are often disappointing because of the lack of relevant differentiation factors that could drive MSC differentiation towards a stable mature chondrocyte phenotype.

**Results:**

We used a large-scale transcriptomic approach to identify genes that are modulated at early stages of chondrogenic differentiation using the reference cartilage micropellet model. We identified several modulated genes and selected neuromedin B (NMB) as one of the early and transiently modulated genes. We found that the timely regulated increase of NMB was specific for chondrogenesis and not observed during osteogenesis or adipogenesis. Furthermore, NMB expression levels correlated with the differentiation capacity of MSCs and its inhibition resulted in impaired chondrogenic differentiation indicating that NMB is required for chondrogenesis. We further showed that NMB activated the calcineurin activity through a Ca^2+^-dependent signaling pathway.

**Conclusion:**

NMB is a newly described chondroinductive bioactive factor that upregulates the key chondrogenic transcription factor Sox9 through the modulation of Ca^2+^ signaling pathway and calcineurin activity.

**Graphical abstract:**

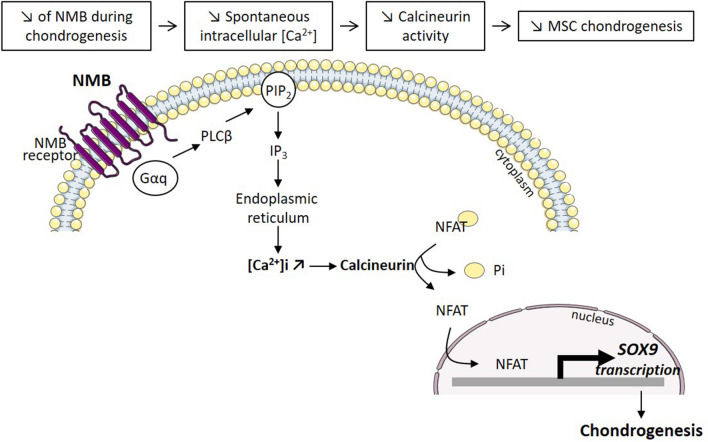

**Supplementary Information:**

The online version contains supplementary material available at 10.1186/s13578-021-00695-1.

## Introduction

Articular cartilage is located at the extremities of long bones and is essential for frictionless joint movement. Chondrocytes are the only mature cells found in this tissue, whose function is to secrete the extracellular matrix (ECM). The ECM is mainly composed of proteoglycans and collagens, which confer to cartilage its viscoelastic and mechanical properties. However, articular cartilage has little capacity for self-repair and cannot regenerate after severe joint injuries or advanced osteoarthritis. Common surgical treatments use chondroplasty, microfracture and mosaicplasty while more modern cartilage repair strategies include matrix-assisted chondrocyte implantation or autologous matrix induced chondrogenesis [1]. Cartilage engineering approaches based on mesenchymal stromal/stem cells (MSCs) have provided promising results in pre-clinical models when MSCs are used in combination with scaffolds and differentiation factors such as transforming growth factor β (TGFβ) [2]. Yet, cartilage repair procedures may be disappointing on the longer term and lead to a non-functional fibrous or hypertrophic cartilage tissue. These results might be explained by the inclusion of patients with lower probability of successful repair (large lesions, older age, inflammation, comorbidity…) and/or the lack of a relevant differentiation factor that could be used to drive MSC differentiation towards a stable mature chondrocyte phenotype.

Chondrogenesis is a complex process in which, MSCs differentiate into chondrocytes under the spatiotemporal control of different growth and transcription factors or microRNAs [3]. However, a complete understanding of the molecular mechanisms controlling differentiation is still missing. Several transcriptomic analyses have been performed on MSCs, embryonic stem cells or induced pluripotent stem cells that were differentiated towards chondrocytes. Many microRNAs, long non-coding RNAs and mRNA transcripts have been found to be modulated during the differentiation process [4–14]. In addition, some gene transcripts have been reported to be predictive markers of high differentiation potential. As examples, miR-210 and miR-630 were shown to be positive regulators of chondrogenesis, and the expression levels of cyclin-dependent kinase 4 inhibitor 2D (CDKN2D), CD74, and transglutaminase 2 (TGM2) to be tenfold higher in highly potent MSCs [15, 16]. In the different studies, the primary model used was the micropellet culture model, obtained after aggregation and culture of stem cells in a chondrogenic medium containing TGFβ3 during 3 weeks [17]. Gene expression was compared between chondrocyte samples (TGFβ3-induced micropellets, different time points) and MSCs (day 0, before aggregation) but never with MSCs cultured in micropellets (3D culture conditions in absence of chondro-inductive factors).

The aim of the present study was to identify new genes involved in the early steps of chondrogenesis by large-scale transcriptomic analysis. This approach should identify genes governing the initiation of chondrocyte differentiation, and genes predicting MSCs with high chondrogenic potential. The originality of our approach was to exclude the genes modulated by the 3D culture conditions, thereby highlighting the genes modulated during the chondrogenic differentiation of MSCs.

## Results

### Global gene analysis in the early stages of chondrogenesis

**Fig. 1:**
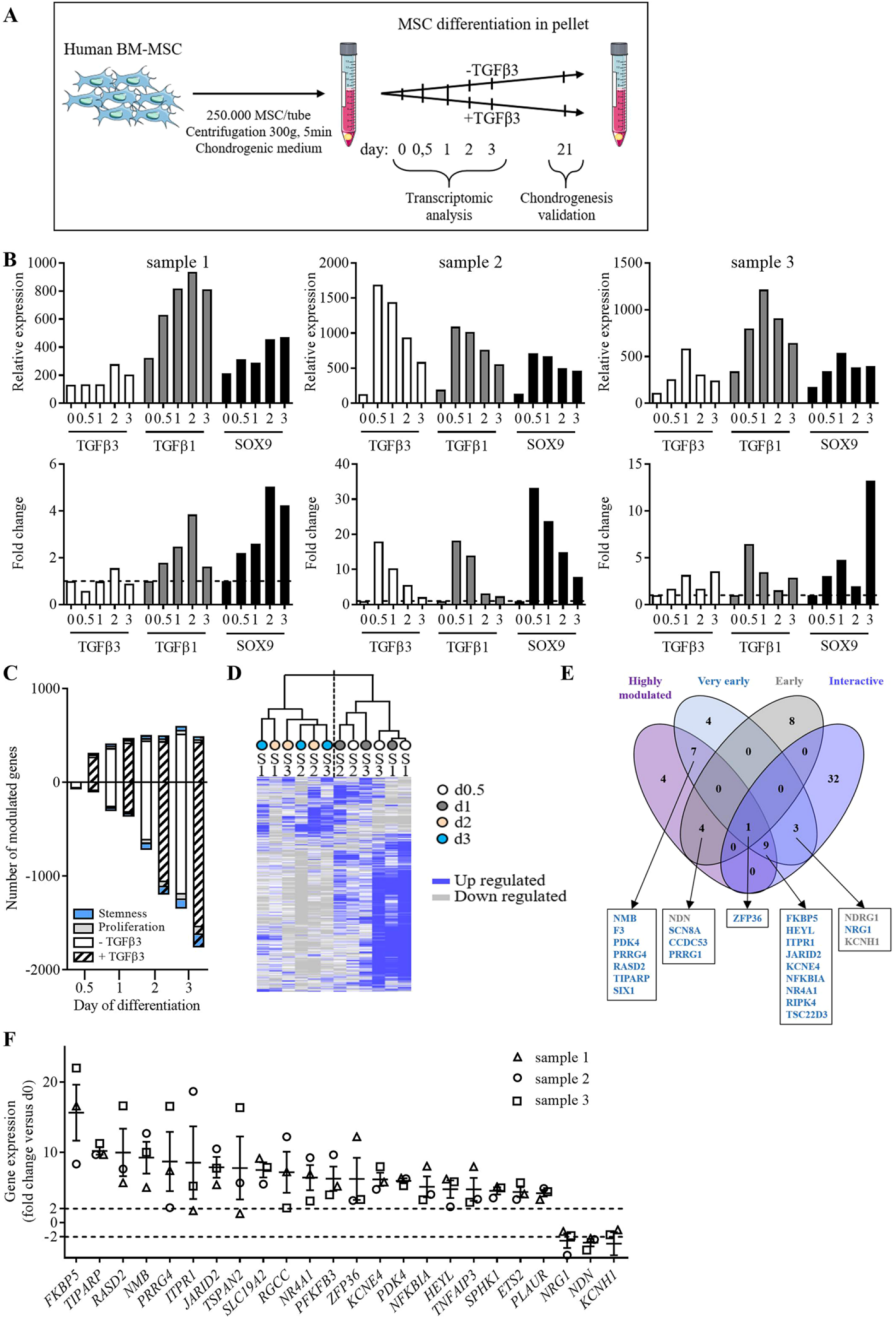
Microarray analysis and gene selection.** A** Experimental work flow. Bone-marrow MSCs (BM-MSC) were induced to differentiate into chondrocytes using the pellet culture conditions with or without TGFβ3. Pellets were recovered at different time points (day 0, 0.5, 1, 2 and 3) for transcriptomic analysis and at d21, for validating chondrogenic differentiation. **B** Fold change expression of 3 selected genes in the 3 samples used in microarrays as determined by the transcriptomic analysis (upper panel) or by real-time qPCR (bottom panel). **C** Number of significantly up-regulated (positive values) and down-regulated (negative values) genes at different time points as compared to d0, in samples differentiated without (blank bar) or with TGFβ3 (hatched bar). The number of genes relative to proliferation or stemness signature are in grey and blue, respectively. **D** Unsupervised hierarchical clustering. Gene expression for the 3 BM-MSC samples (S1-3) cultured in TGFβ3 condition, at different time points (d0.5, 1, 2, 3) using the 17,000 probe sets. Up regulated and down regulated genes are in blue and grey colors, respectively. **E** Venn diagram including the top 25 up- and down-regulated genes independently of the time points (called “most regulated”, in purple), the genes significantly regulated at d0.5 and d1 (called “very early genes”, in blue), the genes significantly regulated at d0.5 and d1 (called “early genes”, in grey) and the genes significantly regulated at the 4 time points and interacting within the same pathway as determined by IPA analysis (called “interactive”, in dark purple). Up- and down-regulated selected genes are in blue and grey, respectively. **F** Relative expression of the genes selected in (**E**) as determined by the transcriptomic analysis

In order to identify new genes involved in the early steps of chondrogenesis, we performed a transcriptomic analysis on 3 independent samples of human bone marrow MSCs (BM-MSCs). BM-MSCs were induced to differentiate toward chondrocytes using the micropellet culture conditions in presence of TGFβ3. To minimize the modulation of gene expression induced by the culture of MSCs from 2 to 3D, we compared the genes expressed by micropellets in presence of TGFβ3 (chondrogenic condition) and in absence of TGFβ3 (3D condition) at different time points (day 0, 0.5, 1, 2 and 3) (see the workflow in Fig. 1A). Chondrogenic differentiation of the three samples was confirmed at day 21 by increased expression of chondrocyte markers (data not shown). At each time point and in each sample, the gene expression was normalized to day 0 and compared between the two conditions (chondrogenic vs 3D). To validate our analysis, we examined the expression of three genes, *TGFβ3*, *TGFβ1* and SRY-box transcription factor 9 *(SOX9)* that are known to be increased at early stages of chondrogenesis. Quite similar gene expression kinetic profiles were obtained in the microarrays (upper panel) and by RT-qPCR analysis (bottom panel) for the three samples suggesting the validity of our transcriptomic data (Fig. 1B).

Using the Venny tool, we excluded the genes that were specific for and/or common to the 3D condition and found 1,929 genes that were specific for the chondrogenic condition and significantly modulated compared to day 0. Global analysis of the genes that were differentially modulated at different time points as compared to day 0 revealed a higher number of genes up-regulated at day 0.5 and 1 and a higher number of genes down-regulated at day 2 and 3 (78% of all modulated genes at day 3) (Fig. 1C). The number of up-regulated genes remained stable from day 1 to day 3 while the down-regulated genes constantly increased. Similar profiles were obtained for the two culture conditions (chondrogenic and 3D). Day 0.5 appeared as an essential time point because the up-regulated genes were only observed in the samples cultured in the chondrogenic condition. Interestingly, using a list of genes assigned to stemness or proliferation (see Section "Method"), we noticed that only a small proportion of modulated genes were involved in these processes suggesting that most of the genes might be related to the initiation of differentiation. For the following analyses, the genes common to the chondrogenic and 3D conditions were excluded as being related to the 3D culture and not to chondrogenesis.

Hierarchical clustering based on the transcripts differentially expressed in micropellets cultured under the chondrogenic condition highlighted a segregation of samples according to the time points: the very early modulated genes (days 0.5–1) and the early modulated genes (days 2–3) (Fig. 1D). Looking at the most highly regulated genes at the different time points, we identified many genes that were already known to be involved in chondrogenesis and/or expressed in chondrocytes according to the literature (data not shown). These genes were excluded and the remaining genes were attributed to subgroups. We classified a group of the most highly modulated genes (first 25 genes), or the group of very early genes (32 genes with a fold change value > 2), or the group of early genes (13 genes with a fold change value > 2) or the so-called interactive group, which included genes that were modulated in these last two groups and reported to interact in a same pathway, as determined using a Gene Ontology analysis. A Venn diagram encompassing the genes significantly modulated (with a p-value < 0.05) summarized this analysis (Fig. 1E). Only one gene, zing finger protein 36 (*ZFP36)*, was found expressed in the four groups while nine genes were expressed in three groups and twenty-two genes were common in two groups. At the end, twenty-four genes were retained as being specific for the chondrogenic condition and significantly modulated in at least two groups (Fig. 1F). Among the twenty-four genes selected, twenty-one genes were up-regulated and only three were down-regulated.

### Identification of NMB as specifically upregulated during chondrogenesis

**Fig. 2:**
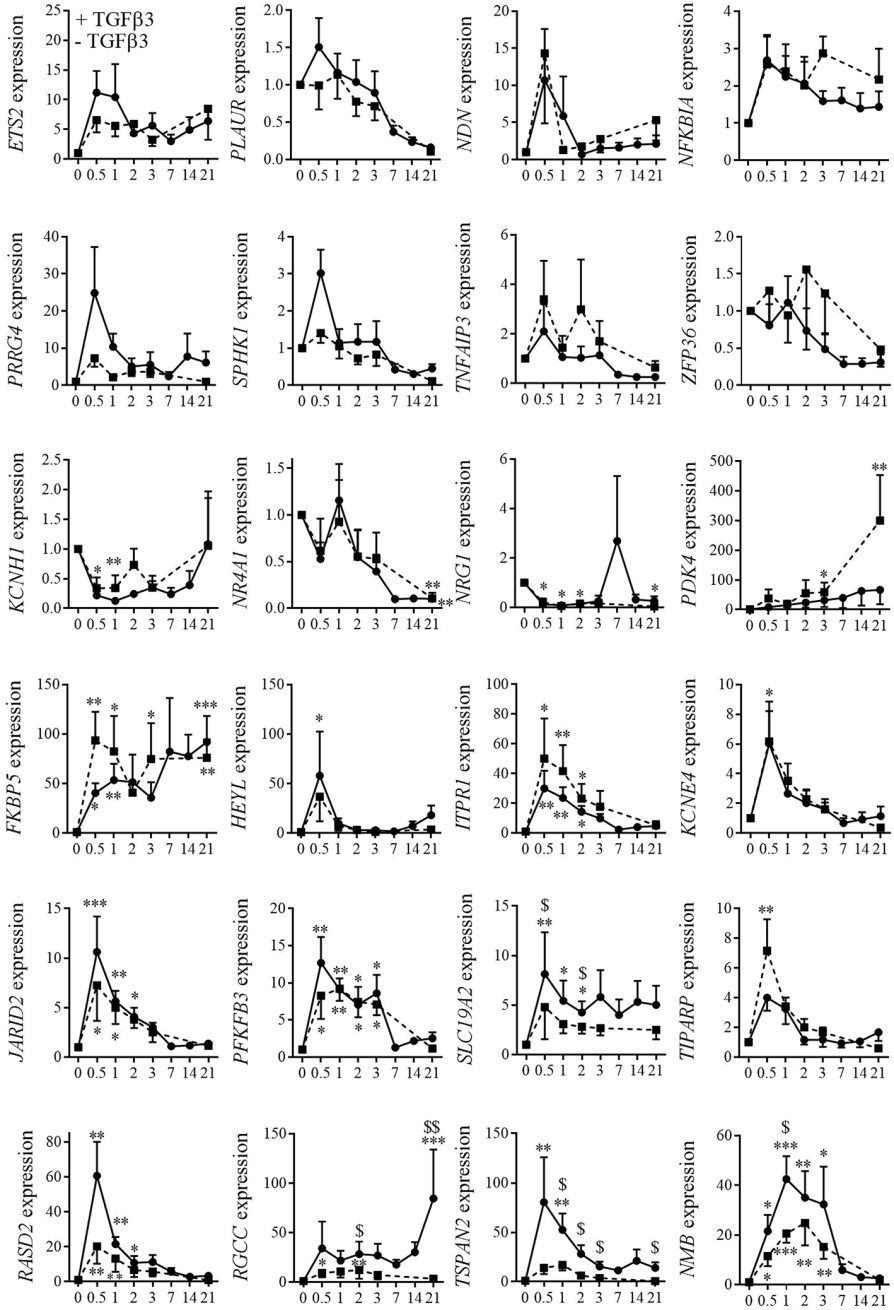
Expression kinetics of selected genes during chondrogenesis. Expression of selected genes were quantified at different time point in 6 individual BM-MSC replicates during chondrogenesis in pellet conditions with (black line) or without TGFβ3 (hatched line) by RT-qPCR. Results are normalized to the expression at day 0 and expressed as the mean ± sem. Statistical significance of gene expression was indicated as *: p ≤ 0.05, **: p ≤ 0.01, ***: p ≤ 0.001 when compared to d0 and as ^$^: p ≤ 0.05, ^$$^: p ≤ 0.01 when samples cultured + or − TGFβ3 were compared. (*HEYL*: Hes Related Family BHLH Transcription Factor with YRPW Motif Like; *PDK4* (Pyruvate Dehydrogenase Kinase 4); *TIPARP* (TCDD Inducible Poly(ADP-Ribose) Polymerase); *NDN* (Necdin, MAGE Family Member); *ZFP36* (ZFP36 Ring Finger Protein); *TNFAIP3* (TNF Alpha Induced Protein); *KCNH1* (Potassium Voltage-Gated Channel Subfamily H Member 1); *FKBP5* (FKBP Prolyl Isomerase 5); *PRRG4* (Proline Rich And Gla Domain;) *ETS2* (ETS Proto-Oncogene 2, Transcription Factor); *JARID2* (Jumonji And AT-Rich Interaction Domain Containing 2); *ITPR1* (Inositol 1,4,5-Trisphosphate Receptor Type 1); *SLC19A2* (Solute Carrier Family 19 Member 2); *NRG1* (Neuregulin 1); *PFKFB3* (6-Phosphofructo-2-Kinase/Fructose-2,6-Biphosphatase 3); *SPHK1* (Sphingosine Kinase 1); *NR4A1* (Nuclear Receptor Subfamily 4 Group A Member 1); *PLAUR* (Plasminogen Activator, Urokinase Receptor); *KCNE4* (Potassium Voltage-Gated Channel Subfamily E Regulatory Subunit 4); *NFKBIA* (NFKB Inhibitor Alpha); *TSPAN2* (Tetraspanin 2); *RASD2* (RASD Family Member 2); *RGCC* (Regulator of Cell Cycle); *NMB* (Neuromedin B))

To validate the twenty-four genes selected from the transcriptomic analysis, six new samples of BM-MSCs were induced to differentiate toward chondrocytes using the micropellet culture conditions in presence or in absence of TGFβ3. A kinetic study of mRNA expression during chondrogenesis revealed that eight genes (*ETS2, PLAUR, NDN, NFKBIA, PRRG4, SPHK1, TNFAIP3, ZFP36)* were not differentially modulated between the chondrogenic and 3D conditions (Fig. 2). The expression of three genes (*KCNH1, NR4A1, NRG1)* was significantly decreased compared to day 0 whatever the culture conditions. Ten genes (*PDK4*, *FKBP5*, *HEYL*, *ITPR1*, *KCNE4*, *JARID2*, *PFKFB3*, *SLC19A2*, *TIPARP, RASD2*) were significantly up-regulated compared to day 0 but with a fold change less than 10 or not significantly different from the 3D condition. Finally, three genes (*RGCC, TSPAN2*, *NMB)* were selected because they were up-regulated more than 40-fold at days 0.5 or 1 and their expression was significantly higher in the chondrogenic condition than in the 3D condition in at least one-time point.

**Fig. 3:**
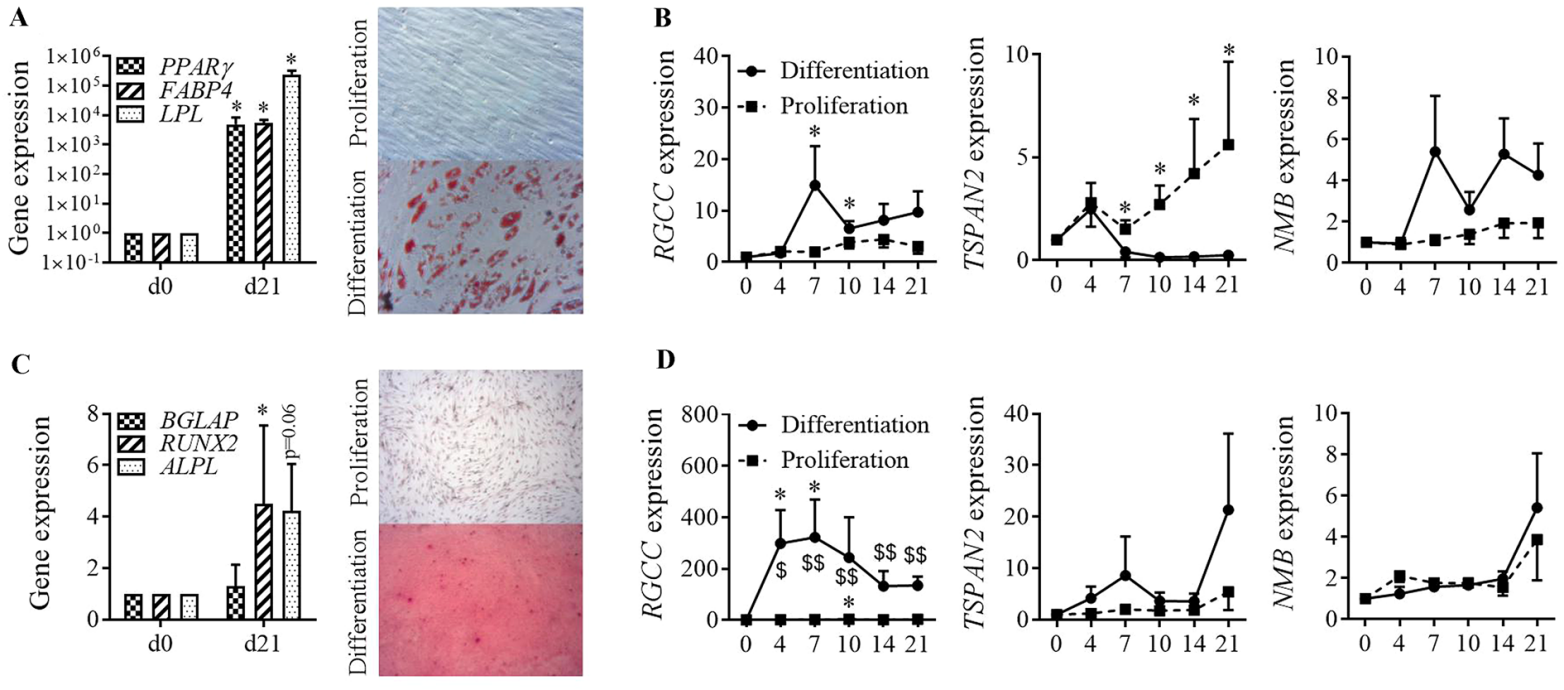
Relative gene expression during osteogenesis and adipogenesis.** A** Adipogenic differentiation of BM-MSCs characterized by increased expression of peroxysome proliferator-activated receptor (*PPAR)-γ*, Fatty Acid Binding Protein (*FABP4*) and lipoprotein lipase (*LPL*) and by staining of lipid droplets by Red Oil O in differentiation versus proliferative conditions at day 21. **B** Relative expression of *RGCC*, *TSPAN2* and *NMB* at different time points during adipogenic differentiation (black line) or in proliferative conditions (hatched line). **C** Osteogenic differentiation of BM-MSCs characterized by increased expression of *RUNX2*, osteocalcin (*BGLAP*), alkaline phosphatase (*ALPL*) and by Alizarin Red S staining in differentiation versus proliferative conditions at day 21. **D** Relative expression of *RGCC*, *TSPAN2* and *NMB* at different time points during osteogenic differentiation (black line) or in proliferative conditions (hatched line). Results are expressed as mean fold change ± sem and statistical significance was indicated as *: p ≤ 0.05, **: p ≤ 0.01, ***: p ≤ 0.001 when samples were compared to day 0 and as ^$^: p ≤ 0.05, ^$$^: p ≤ 0.01 when proliferation and differentiation conditions were compared (n = 6 biological replicates)

The specificity of expression of these factors during chondrogenesis was evaluated on 3 independent samples of BM-MSCs induced to differentiate toward adipocytes or osteoblasts. After 21 days in adipogenic medium, the adipocyte markers (*PPARγ*, *FABP4*, *LPL*) were significantly up-regulated and MSCs were stained by Oil red O indicating the storage of triglycerides in lipid droplets (Fig. 3A). No differentiation occurred in the proliferation medium. At different time points during adipogenesis, the expression of the three genes was quantified. The expression of *RGCC* was significantly increased at days 7 and 10 while the expression of *TSPAN2* was increased after day 7 only in the proliferation condition (Fig. 3B). The expression of *NMB* did not change during the course of adipogenesis. After 21 days in osteoblastogenic medium, the osteogenic markers (*BGLAP*, *RUNX2*, *ALPL*) were significantly upregulated and mineralization was demonstrated by Alizarin Red S staining (Fig. 3C). During osteoblastogenesis, the expression of *RGCC* increased by more than 200-fold from day 4 till day 21 while the expression of *TSPAN2* and *NMB* was not modulated (Fig. 3D). Altogether, we found that the two genes *TSPAN2* and *NMB* were specifically modulated during chondrogenesis only. *TSPAN2* is a plasma membrane associated protein while *NMB* is a secreted factor. We therefore focused our attention on *NMB*, whose soluble form might allow to induce chondrogenesis in a more controllable way.

### Correlation between the expression of NMB and chondrocyte markers

**Fig. 4:**
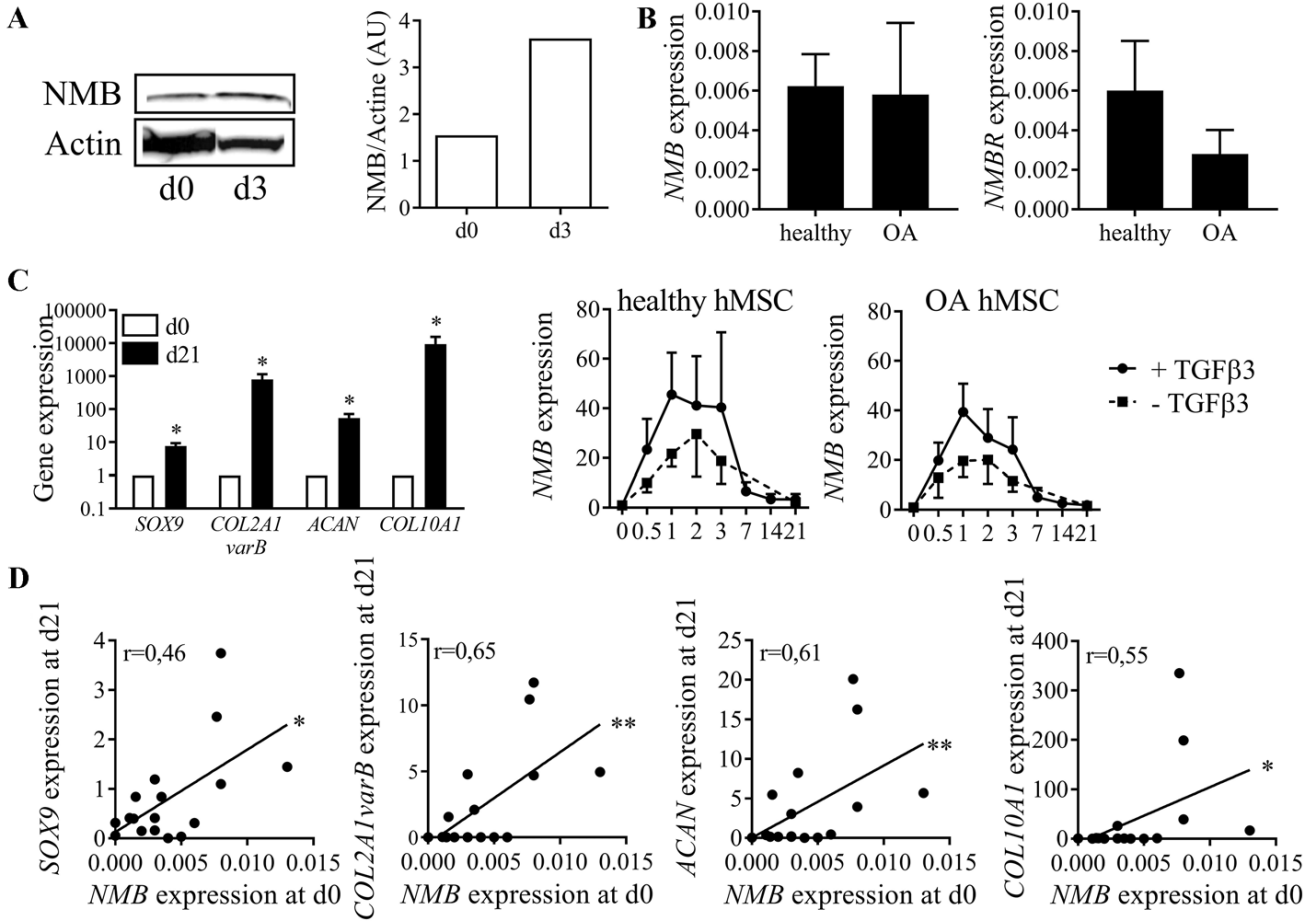
NMB expression during chondrogenesis.** A** Detection of NMB (13 KDa) and β-actin (45 KDa) proteins in BM-MSCs at d0 and micropellets at d3 by Western blotting (left panel) and their respective quantification (right panel). **B** Relative expression of *NMB* and *NMBR* in BM-MSCs from healthy and OA donors quantified by RT-qPCR. Results are expressed as mean ± sem (n = 3 biological replicates). **C** Chondrogenic differentiation of BM-MSCs characterized by increased gene expression of *SOX9*, collagen type IIB (*COL2A1varB*), aggrecan (*ACAN*) and collagen type X (*COL10A1*) at d21 compared to d0 (left panel) (n = 6 biological replicates). Kinetics of *NMB* expression during chondrogenesis of BM-MSCs isolated from healthy (middle panel) or OA (right panel) donors and cultured with or without TGFβ3 (n = 3 biological replicates by group of donors). **D** Correlation between *NMB* expression in BM-MSCs at d0 and chondrogenic markers (*SOX9*, *COL2A1varB*, *ACAN*, *COL10A1*) at d21 of chondrogenic differentiation (n = 17). *: p ≤ 0.05, **: p ≤ 0.01

We investigated the expression of NMB at the protein level during chondrogenesis and found a higher amount of NMB at day 3 compared to day 0, confirming its upregulation at an early chondrogenic stage (Fig. 4A). We then determined whether *NMB* expression was differentially regulated in MSC samples from osteoarthritis (OA) patients, for which chondrogenesis is known to be impaired. We showed that *NMB* and its receptor *NMBR* were expressed at low but similar levels in BM-MSCs with no difference when BM-MSCs were isolated from healthy or OA donors (Fig. 4B). BM-MSC samples from both healthy and OA subjects were then induced to differentiate into chondrocytes that expressed all the specific markers *SOX9*, *COL2A1 variant B*, *COL10A1*, and *ACAN* after 21 days (Fig. 4C). Interestingly, the expression of *NMB* was upregulated at early time points of chondrogenesis (from day 0.5 to 3) in BM-MSCs independently of the origin of BM-MSCs, healthy or OA. We also found out that the basal expression of *NMB* in BM-MSCs at day 0 was correlated with the expression level of all tested chondrocyte markers *SOX9*, *COL2A1 variant B*, *ACAN*, *COL10A1* at day 21 (Fig. 4D). Similar correlation was found between NMB and chondrocyte markers at day 3 but no correlation between *NMB* and adipogenic or osteogenic markers was observed (data not shown). We therefore confirmed the upregulation of *NMB* during the first 3 days of chondrogenesis and reported its prognostic value for high chondrogenic differentiation capacity of BM-MSCs.

### Expression of NMB is required for chondrogenesis

**Fig. 5:**
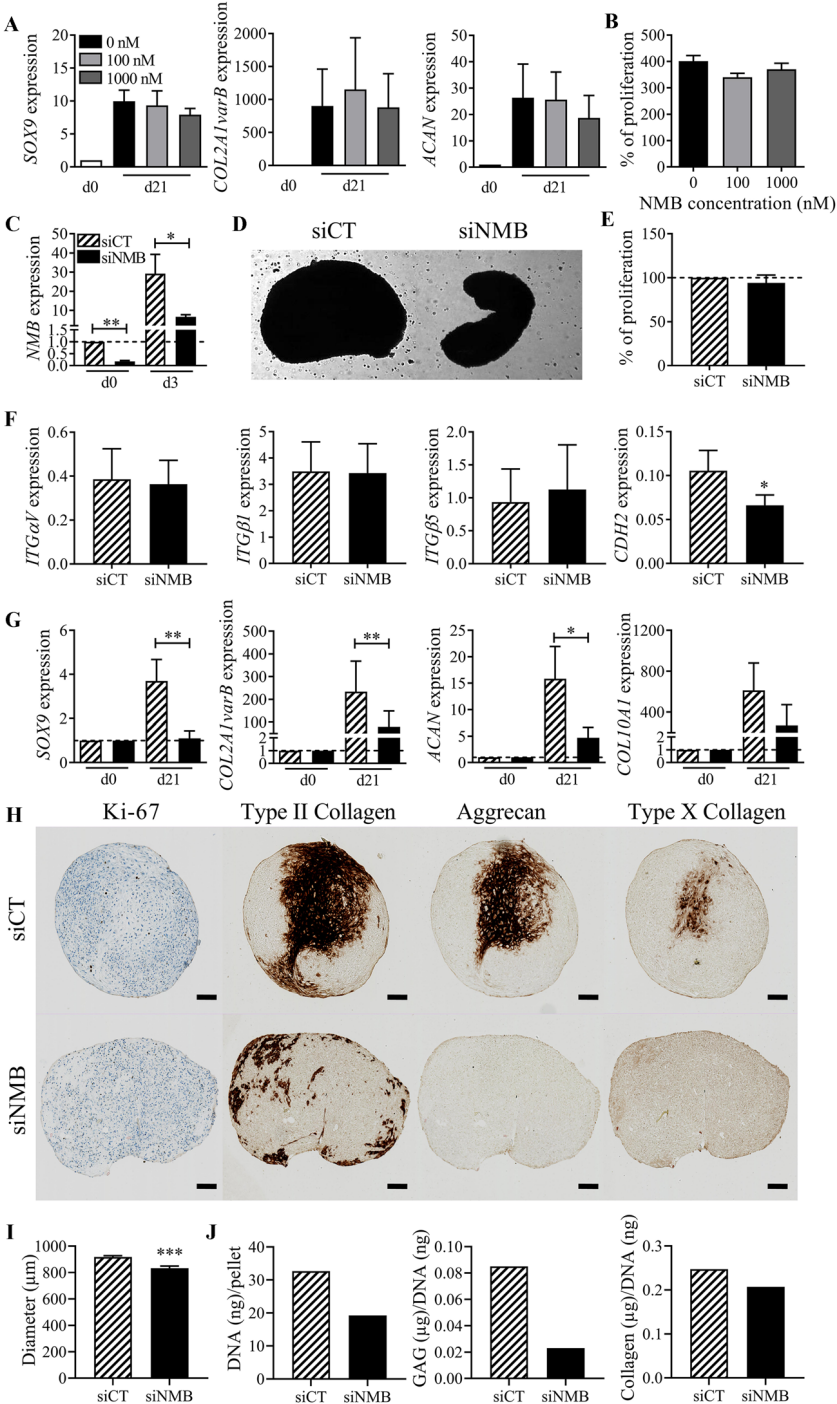
Role of NMB during chondrogenesis. **A** Fold change expression of chondrocyte markers after 21 days of chondrogenic differentiation of BM-MSCs with different concentrations of recombinant NMB. Results are expressed as mean fold change ± SEM (n = 3 biological replicates). **B** Proliferation of BM-MSCs cultured with different concentrations of recombinant NMB after 5 days of culture. Results are expressed as the mean percentage ± sem (n = 4 biological replicates). **C** Fold change expression of NMB in BM-MSCs at day 3 after transfection with siRNA control (siCT) or siRNA against NMB (siNMB) (normalized to NMB expression at d0). **D** Representative picture of micropellets after 3 days of chondrogenic differentiation of BM-MSCs transfected with siCT or siNMB. **E** Proliferation of BM-MSCs transfected with siCT or siNMB after 3 days of culture in monolayer. Results are expressed as mean percentage ± sem (n = 4 biological replicates). **F** Relative expression of adhesion molecules in BM-MSCs transfected with siCT or siNMB after 3 days of chondrogenic differentiation. **G** Fold change expression of chondrocyte markers in BM-MSCs transfected with siCT or siNMB at day 21 of chondrogenesis. Results are expressed as mean ± sem (n = 8 biological replicates). *: p ≤ 0.05, **: p ≤ 0.01. **H** Immunohistological staining of Ki-67, type II Collagen, Aggrecan and type X Collagen in a micropellet representative of BM-MSCs transfected with siCT or siNMB at day 21 of chondrogenesis (scale bar = 100 µm). **I** Average diameter of micropellets from BM-MSCs transfected with siCT or siNMB at day 21 of chondrogenesis (n = 15). **J** Amounts of total DNA, GAG and collagen contained in micropellets of BM-MSCs transfected with siCT or siNMB at day 21 of chondrogenesis (pool of 5 pellets)

Because NMB expression is correlated with high differentiation capacity, we tested the impact of exogenously added recombinant NMB (rNMB) on the differentiation of BM-MSCs. Compared to control BM-MSCs cultured in micropellets with TGFβ3, the addition of increasing doses of rNMB did not modulate the expression of chondrocyte markers (*SOX9*, *COL2A1* variant B, and *ACAN*) by day 21 (Fig. 5A). We checked whether the lack of inductive effect of rNMB could be related to a possible role on proliferation. The addition of rNMB (100 or 1000 nM) during the expansion of BM-MSCs for 5 days did not impact their proliferation compared to the untreated control (Fig. 5B). We then evaluated whether the down-regulation of NMB could interfere with TGFβ3-induced chondrogenesis, using a RNA interference approach. BM-MSCs were transfected twice with a siRNA targeting NMB (siNMB) or a control siRNA (siCT). The transfection of siNMB inhibited the expression of *NMB* by 80% and 77% at day 0 and day 3, respectively (Fig. 5C). Macroscopically at day 3, the micropellets obtained with siNMB-transfected BM-MSCs did not form a homogenous round/ovoid shape and were more loosen than those from control cells (Fig. 5D). The down-regulation of NMB was likely not related to an inhibitory effect on MSC proliferation (Fig. 5E) but we observed a significant inhibition of the adhesion molecule *CDH2* in siNMB-transfected BM-MSCs (Fig. 5F). No impact on the expression of other adhesion molecules *ITGαυ*, *ITGβ1* and *ITGβ1* was noticed. Interestingly, the downregulation of NMB resulted in the downregulation of the chondrocyte markers *SOX9, COL2A1* variant B*, ACAN and COL10A1* at day 21 (Fig. 5G). The downregulation of the chondrocyte markers was confirmed at the protein level. Immunostaining of type II and X collagens as well as aggrecan was reduced in micropellets of siNMB-transfected BM-MSCs compared to siCT-transfected BM-MSCs (Fig. 5H). Few proliferative Ki-67^+^ cells were observed in both siCT- and siNMB-transfected micropellets. Finally, the diameter of micropellets from siNMB-transfected BM-MSCs as well as the GAG, collagen and DNA contents were also reduced in comparison with siCT-transfected BM-MSCs (Fig. 5I–J). These results indicated that NMB acts in an autocrine manner in MSCs and its expression is necessary but not sufficient for the induction of chondrogenic differentiation.

### NMB regulates Sox9 expression through PLC signaling and calcium release at early steps of chondrogenesis

**Fig. 6:**
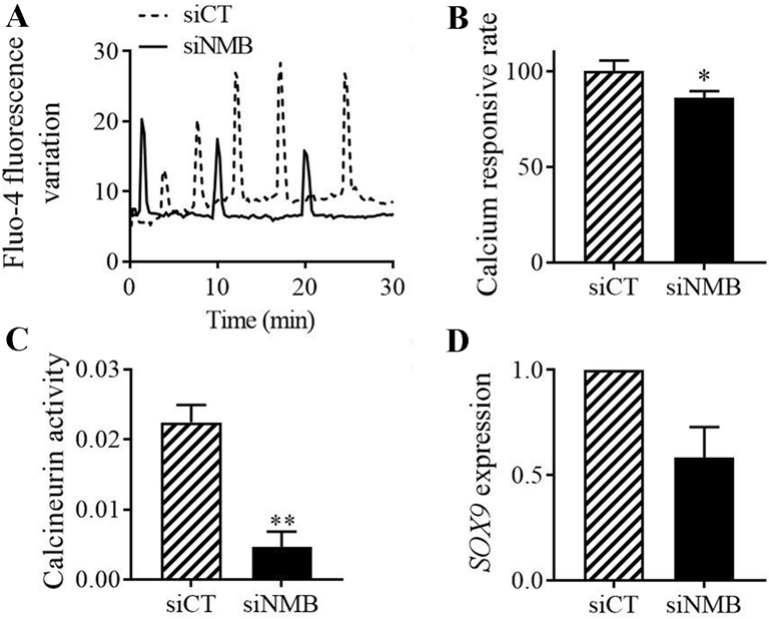
Role of NMB in calcium signaling. **A** Time course of maximal calcium intensity of spontaneous calcium oscillations in BM-MSCs transfected with siCT or siNMB and loaded with Fluo-4-AM at day 3 of chondrogenesis. **B** Calcium responsive rate determined in BM-MSCs transfected with siCT or siNMB at day 3 of chondrogenesis and expressed as fold change rate. **C** Intracellular calcineurin phosphatase activity determined in BM-MSCs transfected with siCT or siNMB at day 3 of chondrogenesis. **D** Fold change expression of *SOX9* in BM-MSCs transfected with siCT or siNMB at day 3 of chondrogenesis. *: p ≤ 0.05, **: p ≤ 0.01

Multiple intracellular signaling pathways are activated upon binding of NMB to its receptors. One of these includes the hydrolysis of phosphatidyl inositol 4,5-biphosphate (PIP_2_) by the isoform β of phospholipase C (PLC_β_) into 1,2-diacylglycerol (DAG) and inositol 1,4,5-triphosphate (iP_3_). Binding of iP_3_ to its receptors in the endoplasmic reticulum triggers the release of Ca^2+^ in the cytosol [18]. Because the increase of Ca^2+^-influx was previously reported to be indispensable for proper chondrogenesis [19], we investigated the intracellular calcium concentration in siNMB-transfected MSCs induced to differentiate toward chondrocytes. The spontaneous calcium oscillations were determined in Fluo-4-AM loaded BM-MSCs at day 3 of differentiation when NMB levels were high. A representative time course of maximal Ca^2+^ intensity revealed both a lower number and a lower maximal intensity of Ca^2+^ flux pics in micropellets of siNMB-transfected BM-MSCs compared to control micropellets (Fig. 6A). This result was confirmed by quantifying the Ca^2+^ responsive rate, which was lower in micropellets of siNMB-transfected BM-MSCs (Fig. 6B). We also determined the levels of cellular calcineurin phosphatase activity, which is dependent of Ca^2+^ concentration and positively regulates chondrogenesis [19]. At day 3 of chondrogenesis, the calcineurin activity was significantly reduced in micropellets of siNMB-transfected BM-MSCs compared to siCT-transfected BM-MSCs, which was related with the inhibition of *SOX9* expression (Fig. 6C–D). Taken together, these results suggest that NMB is the key player of intracytoplasmic Ca^2+^ regulation resulting in successful chondrogenesis.

## Discussion

In the present study, we highlighted an inducting role of NMB in the early phases of MSC differentiation towards chondrocytes that has not been previously described. We made the link between the transient upregulation of NMB during the first days of chondrogenesis and the modulation of Ca^2+^ signaling pathway that is essential for optimal differentiation.

With 3 biologic replicates and four time points during the first 3 days of chondrogenic differentiation of MSCs, our data present an in-depth analysis of the gene expression changes induced during MSC chondrogenesis. The analysis provided a list of genes already known to be modulated during chondrogenesis (data not shown) and a set of genes not previously described as associated with chondrogenesis. The main difference of our analysis with other large-scale transcriptomic analyses is the identification of genes that were modulated during the chondrogenic differentiation of MSCs, but not related to the changes from 2 to 3D culture conditions [11, 13, 14]. This allowed to identify several genes that might be important regulators of chondrogenesis. We focused our attention on NMB, a secreted factor that might be clinically use as an exogenous agent for MSCs differentiation, without genetic engineering.

The importance of the intracellular Ca^2+^ modulation at the onset of chondrogenic differentiation has been shown using high-density cultures of chicken limb bud-derived mesenchymal cells [19]. In this study, the basal level of Ca^2+^ was reported to increase on day 3 of differentiation. The changes of cytosolic free Ca^2+^ concentration were not dependent on intracellular Ca^2+^ stores but rather largely dependent on extracellular sources. Interestingly, slight and transient elevation of cytosolic Ca^2+^ levels promoted differentiation through the regulation of cell proliferation while high increase of Ca^2+^ inhibited chondrogenesis. In line with our results where NMB is transiently upregulated during the first days of chondrogenesis, the authors demonstrated that transient elevation of Ca^2+^ preceded differentiation and manipulation of intracellular concentration of calcium altered chondrogenesis. The signaling molecule in this process was calcineurin, whose role in the regulation of intracellular Ca2^+^ influx was crucial. A previous study described the positive role of calcineurin in the phosphorylation of SOX9 and chondrogenesis regulation [20]. Ca^2+^ influx-dependent SOX9 activation was also reported during chondrogenesis of C3H10T1/2 MSCs and ATDC5 chondroprogenitor cells [21]. In these cells, the activation of SOX9 by transient receptor potential vanilloid 4 (TRPV4), a calcium channel involved in Ca^2+^/calmodulin signaling induced GAG accumulation when applied with other inductive signals such as insulin or BMP-2 and contributed to the process of chondrogenesis. Other types of calcium channels, the voltage gated Ca^2+^ channels (VGCC) are also key regulators in chondrocytes. Of interest, Ca^2+^ influx via the Ca_V_3.2 VGCC activates the calcineurin/nuclear factor of the activated T-cell (NFAT) signaling pathway and a NFAT binding site has been identified within the SOX9 promoter [22]. Different types of calcium channels have been identified in chondrocytes, including VGCC, TRP, acid-sensing ion channels as discussed in a recent review on the chondrocyte channelome [23]. The role of these channels in chondrogenesis is still imperfectly understood. However, the importance of Ca^2+^ influx from the extracellular compartment and the activation of the calcineurin/NFAT signaling pathway leading to SOX9 activation has been demonstrated in chondrocytes [19].

In addition to extracellular Ca^2+^ influx through plasma membrane associated channels, transient increase of cytosolic Ca^2+^ can occur through the release of Ca^2+^ from endoplasmic reticulum (ER) stores. Rapid depletion of ER Ca^2+^ stores activates Ca^2+^ entry across the plasma membrane, a process called store-operated Ca^2+^ entry (SOCE), which has been reported in chondrocytes and chondroprogenitor cells [19, 24]. In this process, phospholipase C (PLC) increases inositol phosphate levels and induces cytosolic release of intracellular Ca^2+^ from ER stores. Of interest, the PLC signaling pathway is activated by NMB upon binding to its receptors in various NMBR-expressing cells [18]. The signaling cascade results in DNA synthesis and cell proliferation in a number of tumor cells [25, 26]. In MSCs, the modulation of NMB expression did not impact their proliferation but the inhibition of NMB resulted in lower Ca^2+^ and calcineurin activity, and reduced *SOX9* expression. We therefore provide evidence that NMB-induced intracellular Ca^2+^ influx induces SOX9-dependent chondrogenesis through the activation of calcineurin/NFAT pathway, probably via PLC activation (Graphical abstract). These results provide a novel insight on the regulation of Ca^2+^ signaling pathway during chondrogenesis.

Besides their role during chondrogenesis, Ca^2+^ signaling pathways are important components of mechanotransduction pathways in chondrocytes. Indeed, a positive correlation between cartilage extracellular matrix synthesis and Ca^2+^ signaling has been reported under mechanical load [27]. TRPV4 is a positive regulator of SOX9 and mutations in this gene are linked to chondrodysplasia [28]. In addition, TRPV6-knockout mice exhibit severe OA changes suggesting that TRPV6 may be a chondroprotective factor [29]. Accumulating evidence indicates that altered expression or function of Ca^2+^ channels, including TRP channels, might be implicated in OA development [23]. An increase in Ca^2+^ signaling may be one cause of chondrocyte dysfunction in OA suggesting that inhibition of Ca^2+^ channels could be therapeutic targets for OA [30]. Actually, the deletion of TRPV4 reduced the severity of age-related OA development although it did not prevent destabilization of the medial meniscus (DMM)-induced OA development [31]. Our preliminary results indicate that down-regulation of NMB using a siRNA approach in chondrocytes from OA patients results in the up-regulation of chondrocyte markers while the up-regulation of NMB using the NMBR inhibitor BIM reverses this effect (Additional file 1: Fig. S1). These results confirmed that the increase of free cytosolic Ca^2+^ above a threshold level could be detrimental to chondrocyte homeostasis and chondrogenesis [19]. In line with these data, a pilot study in OA patients has revealed that Ca^2+^ antagonists may exert beneficial side clinical effects in preventing the progression of the disease [32]. These results therefore suggest that modulation of Ca^2+^ signaling may counteract the catabolic response and defective synthesis of extracellular matrix molecules in OA cartilage.

## Conclusions

We showed that NMB upregulation is required during the early phases of MSC differentiation towards chondrocytes and made the link between the transient upregulation of NMB and the modulation of Ca^2+^ signaling pathway. A better understanding of the involvement of Ca^2+^ channels and the modulation of Ca^2+^ signaling in chondrocytes under healthy and OA conditions is needed before to investigate novel therapeutic strategies.

## Material and methods

### Cell isolation and culture

Human bone marrow (BM)-MSCs and chondrocytes were recovered from patients after written informed consent as approved by the French Ministry of Higher Education and Research (DC-2009-1052). BM-MSCs were characterized by their immunophenotype CD11b^−^/CD19^−^/CD34^−^/CD45^−^/CD73^+^/CD90^+^/CD105^+^ and trilineage differentiation potential as previously described and used before passage [33]. They were maintained in proliferative medium consisting in α-MEM (Lonza; Levallois-Perret, France), 1 ng/mL of basic fibroblast growth factor (bFGF; R&D Systems; Noyal Châtillon sur Seiche, France), 100 µg/mL penicillin/streptomycin (PS; Lonza), 2 mM glutamine (Lonza) and supplemented with 10% fetal calf serum (FCS). For proliferation assays, BM-MSCs were plated at 10,000 cells/well in 6-well plates in proliferative medium. After 5 days of culture, viable cells were counted after Trypan blue exclusion using a Malassez hemocytometer. BM-MSCs were used between passage 2 and 4.

Cartilage slices (< 1 mm^3^) were recovered from the knee joints of osteoarthritic (OA) patients and incubated in 2.5 mg/mL pronase (Sigma-Aldrich, Saint-Quentin-Fallavier, France) at 37 °C for 1 h followed by an incubation with 2 mg/mL type II collagenase (Sigma) at 37 °C overnight. Digested cartilage tissues were filtrated through a 70 μm cell strainer and chondrocytes were seeded at 25,000 cells/cm^2^ in DMEM high glucose (Lonza) supplemented with 2 mmol/mL glutamine, 100 U/mL PS, 1.25 µg/mL Amphotericin B, 5 µg/mL insulin, 5 ng/mL bFGF and 10% FCS until the end of passage 0.

### Differentiation assays

Chondrogenic differentiation of BM-MSCs was induced by culture in micropellets for 21 days. Briefly, 250,000 cells were pelleted by centrifugation in 15 mL conical tubes (6–8 tubes) and cultured in 3D conditions consisting in DMEM supplemented with 100 μg/mL PS, 0.35 mM proline, 0.1 μM dexamethasone, 0.17 mM ascorbic acid-2-phosphate, 1 mM pyruvate sodium, 1% insulin-transferrin-selenic acid (ITS) (Lonza). In chondrogenic conditions, TGFβ3 (10 ng/mL; R&D Systems) was added extemporaneously. For RNA extraction, micropellets were lyzed in RLT buffer according to the recommendations of the supplier (Qiagen; Les Ulis, France) at different time points (day 0.5, 1, 2, 3, 7, 14 and 21) and stored at −80 °C.

For osteoblastogenesis, BM-MSCs were plated at 3000 cells/cm^2^ and cultured in osteogenic medium (DMEM containing 10% FCS, 10 mM β-glycerophosphate, 0.1 μM dexamethasone, 70 μM ascorbic acid-2-phosphate and 100 μg/mL PS). Cells were either lyzed in RLT buffer (Qiagen) at different time points (day 0.5, 1, 2, 3, 7, 14 and 21) and stored at −80 °C until RNA extraction or fixed with 95% ethanol for Alizarin Red S staining at day 21.

For adipogenic differentiation, BM-MSCs were plated at 9000 cells/cm^2^ and cultured in proliferative medium for 5 days. The differentiation medium (DMEM-F12 containing 5% FCS, 100 μg/mL PS, 16 μM biotin, 18 μM panthotenic acid, 100 μM ascorbic acid, 60 μM indomethacin, 450 μM isobutylmethylxanthine, 1 μM dexamethasone, 1 μM rosiglitazone) was then added and changed every 3 days until day 21. Cells were either lyzed in RLT buffer at different time points (day 0.5, 1, 2, 3, 7, 14 and 21) and stored at −80 °C until RNA extraction or fixed with 2.5% glutaraldehyde for Oil Red O staining at day 21.

### RNA isolation and microarray hybridation

Total RNA was extracted using the RNeasy mini kit (Qiagen) following the supplier’s recommendations. RNA concentration was measured using a Nanodrop (ThermoFisher Scientific; Illkirch, France) and their integrity by using the Agilent 2100 Bioanalyzer (Agilent Technologies). Total RNA (150–200 ng) was reverse-transcribed after ribosomal fraction depletion from 2 µg total RNA, then hybridized onto GeneChip Human Exon 1.0 ST Affymetrix microarray. Ambion WT Expression Kits were used for amplification and Affymetrix WT Terminal Labeling Kits were used for labeling.

### Data processing and gene expression profile analysis

After image processing with the Affymetrix GeneChip® Command Console® Software, the CEL files were analyzed using the Affymetrix Expression Console™ software v1.3.1 to obtain an intensity value signal for each probe set. The probesets were annotated using the Affymetrix annotation file from Netaffx (http://www.netaffx.com). Affymetrix array core probeset data were normalized with the Robust Multiarray Average (RMA) method. Transcripts with significant differential expression profiles were identified using the Significance Analysis of Microarray (SAM) algorithm (http://www-stat.stanford.edu/~tibs/SAM/) with the Wilcoxon test and sample label permutations (n = 300) was used to identify genes of which expression varied significantly between one selected time point (day 0.5, 1, 2 or 3) and day 0. Only transcripts with a fold change (FC) ≥ 2 and significant false discovery rate (FDR) < 5% were retained. Hierarchical clustering was produced using the Cluster and Treeview softwares (PMID: 9843981). The gene ontology (GO) enrichment analysis, the biological processes and networks of differentially expressed genes were analyzed through the use of Ingenuity Pathway Analysis (IPA; QIAGEN Inc., https://www.qiagenbioinformatics.com/products/ingenuitypathway-analysis) (PMID: 24336805). Analysis of the expression of stemness and proliferation-related genes are based on a previously published data set from pluripotent stem cells in which a consensus stemness gene list (n = 1076 genes) was defined (PMID: 17204602) and from proliferating samples including rapidly dividing CD105^+^ endothelial cells, CD71^+^ early erythroid progenitors and cell lines originating from tumors in which a proliferation gene list was defined (PMID: 19128516). Venn diagrams were obtained using Venny tools and represent the number of genes in each comparison and the overlaps between the four comparison groups.

Microarray data are in accordance to Minimum Information About a Microarray Experiment (MIAME) guidelines.

### RT-qPCR analysis

RNA was reverse transcribed using the Moloney Murine Leukaemia Virus Reverse Transcriptase (M-MLV) (Invitrogen, ThermoFisher Scientific) in a non-gradient thermocycler. qPCR was then performed on 10 ng of cDNA using SYBR™ Green I PCR Master Mix (Roche Diagnostics, Meylan, France) or TaqMan™ Universal Master Mix II, with UNG and specific primers (Tables 1, 2). PCR reaction was performed as follow: 95 °C for 5 min; 40 cycles at 95 °C for 15 s; 64 °C for 10 s and 72 °C for 20 s with SYBR Green or 95 °C for 5 min; 40 cycles at 95 °C for 15 s and 64 °C for 10 s with TaqMan, in a ViiA 7 Real-Time PCR System (Applied Biosystems, ThermoFisher Scientific) and analyzed with the dedicated software. All values were normalized to the housekeeping gene *RPS9* and expressed as relative expression using the formulae 2^−∆Ct^ or as fold change using the formulae 2^−∆∆Ct^.

**Table 1:** List of primers for SYBR Green based PCR

Gene name	Sequence forward	Sequence reverse
*ACAN*	TCGAGGACAGCGAGGCC	TCGAGGGTGTAGCGTGTAGAGA
*ALPL*	CCACGTCTTCACATTTGGTG	GCAGTGAAGGGCTTCTTGTC
*BGLAP*	GGCGCTACCTGTATCAATGG	TCAGCCAACTCGTCACAGTC
*CDH2*	CCTCCAGAGTTTACTGCCATGAC	GATGGATCTCCGCCACTGATTC
*COL1A1*	CCTGGATGCCATCAAAGTCT	CGCCATACTCGAACTGGAAT
*COL2A1varB*	CAGACGCTGGTGCTGCT	TCCTGGTTGCCGGACAT
*COL3A1*	CGCCCTCCTAATGGTCAAGG	AGGGCCTGAAGGACCAGCTT
*COL10A1*	TGCTGCCACAAATACCCTTT	GTGGACCAGGAGTACCTTGC
*FABP4*	ATGGGATGGAAAATCAACCA	GTGGAAGTGACGCCTTTCAT
*ITGαυ*	AACTCAAGCAAAAGGGAGCA	GGGTTGCAAGCCTGTTGTAT
*ITGβ1*	TGATGCAGTACTCCAGGCAG	AATCTCCACCGTTGTTCCAG
*ITGβ5*	GCCTTTCTGTGAGTGCGACAAC	CCGATGTAACCTGCATGGCACT
*LPL*	GTCCGTGGCTACCTGTCATT	TGGATCGAGGCCAGTAATTC
*MMP13*	GACTTCCCAGGAATTGGTGA	TACCCCAAATGCTCTTCAGG
*NMBR*	GAAACCCCCACGGAAGTGAG	GGAGTTGGTGATCCGCTGTG
*PPARγ*	CCAGAAAGCGATTCCTTCAC	TGCAACCACTGGATCTGTTC
*RSP9*	ATGAAGGACGGGATGTTCAC	GATTACATCCTGGGCCTGAA
*RUNX2*	CGGAATGCCTCTGCTGTTAT	TTCCCGAGGTCCATCTACTG
*SOX9*	AGGTGCTCAAAGGCTACGAC	GTAATCCGGGTGGTCCTTCT

**Table 2:** List of primers for TaqMan based PCR

Gene name	Primer ID
*ETS2*	Hs01036305_m1
*FKBP5*	Hs01561006_m1
*HEYL*	Hs00232718_m1
*ITPR1*:	Hs00181881_m1
*JARID2*	Hs01004460_m1
*KCNE4*	Hs00298953_m1
*KCNH1*	Hs00924320_m1
*NDN*	Hs00267349_s1
*NFKBIA*	Hs00153283_m1
*NMB*	Hs00963142_m1
*NR4A1*	Hs00374226_m1
*NRG1*	Hs00247620_m1
*PDK4*	Hs01037712_m1
*PFKFB3*	Hs00998700_m1
*PLAUR*	Hs00958880_m1
*PRRG4*	Hs00225378_m1
*RASD2*	Hs00374755_m1
*RGCC*	Hs00204129_m1
*RPS9*	Hs02339424_m1
*SLC19A2*	Hs00949693_m1
*SPHK1*	Hs01116530_g1
*TIPARP*	Hs00296054_m1
*TNFAIP3*	Hs00234713_m1
*TSPAN2*	Hs00194836_m1
*ZFP36*	Hs00185658_m1

### Cell transfection

BM-MSCs were transfected twice: 3 days (80% of confluence) and one day before inducing the chondrogenic differentiation. Transfection was done using 50 nM of predesigned siNMB (TaqMan, siRNA ID: s9582; Applied BIosystems, Illkirch, France) or siRNA control (UAAGGCUAUGAAGAGAUACTT) using Oligofectamine™ in Opti-MEM™ (Life Technologies SAS, Villebon-sur-Yvette, France) during 6 h.

### Cell treatment

BM-MSCs were treated with recombinant NMB (rNMB; peptide sequence: Gly-Asn-Leu-Trp-Ala-Thr-Gly-His-Phe-Mel-NH_2_; Sigma-Aldrich) for the 21 days of chondrogenesis. Two concentrations were evaluated: 100 or 1000 nM and the medium was changed every 3–4 days. Passage 1 chondrocytes were plated at 60,000 cells/well of 6-well plates and treated with the NMBR antagonist BIM23042 (BIM; peptide sequence: D-Nal-Cys-Tyr-D-Trp-Lys-Val-Cys-Nal-NH_2_; Tocris, Noyal Châtillon sur Seiche) at 50 nM during 3 days.

### Western blot

Micropellets were first washed with PBS, then stored in RIPA lysis buffer (Sigma Aldrich) containing a protease inhibitor cocktail (Sigma, P-8340) for 1 h at −80 °C. Micropellets were then grinded and centrifuged at 12,000 rcf, at 4 °C for 15 min. Total proteins (50 µg) diluted in Bolt LDS sample buffer and Bolt Sample Reducing Agent were heated at 70 °C for 10 min and loaded on Bolt 4–12% Bis–Tris Plus precast gels (Life Technologies). Samples were allowed to migrate for 30 min at 200 mV using the MOPS SDS running buffer and transferred on nitrocellulose membranes using the iBlot 2® Gel Transfer Device (Life Technologies). Membranes were then blocked with 5% milk and probed with the anti-human NMB rabbit polyclonal primary antibodies (1:500; Cohesion Biosciences, Nanterre) at 4 °C, overnight. Anti-human Actin mouse polyclonal primary antibodies (1:5000) for 2 h at room temperature. After several rinses with tris phosphate buffered saline (TBS) containing 0.05% tween-20 (Sigma-Aldrich), membranes were incubated with a horseradish peroxidase-conjugated goat anti-rabbit IgG antibody (1:50000) for 1 h at room temperature. Blots were revealed using a WesternBright™ Sirius Chemiluminescent detection kit (Advansta, Blagnac) and scanned (ChemiDoc XRS System, Biorad).

### Immunohistochemistry

Micropellets were fixed in 4% formaldehyde at room temperature for 1 h and processed in paraffin for routine histology. Immunohistochemistry was performed on deparaffinized micropellet Sections (3 μm) using antibodies specific for aggrecan (1/1000 dilution; Chemicon, Millipore, Molsheim), type II collagen (1/50 dilution; Lab Vision, Francheville) and type X collagen (1/50 dilution; Diagomics) and the UltraTek Horse Radish Peroxidase (anti-polyvalent) kit (ScyTek Laboratories) following manufacturer’s instructions. Sections were mounted in Eukitt (Sigma-Aldrich, Lyon). Images were obtained using the slide scanner Hamamatsu Nanozoomer.

### GAG, collagen and DNA quantification

Five micropellets of BM-MSCs transfected with siCT or siNMB were recovered at day 21 of chondrogenesis and digested in 300 µL of papain (592 µg/mL) in a solution of sodium acetate buffer (0.1 M, pH 5.5), EDTA (0.5 M), L-cystein-HCl (5 mM)) at 60 °C during 18 h. Forty microliters were used for GAG quantification, 90 µL for collagen quantification and 60 µL for DNA quantification. GAG, total collagens and DNA were measured using the Glycosaminoglycan Assay Blyscan™ (Biocolor life science assays, Interchim), the Soluble Collagen Assay Sircol™ (Biocolor life science assays, Interchim) and the CyQUANT™ Cell Proliferation Assay (Invitrogen), respectively, following manufacturer’s instructions.

### Analysis of spontaneous calcium transients and calcineurin cellular activity

Spontaneous calcium transients were monitored on micropellets at day 3 of differentiation. Micropellets were rinsed with buffer containing 145 mM NaCl, 5 mM KCl, 2 mM CaCl_2_, 2 mM MgCl_2_, 10 mM HEPES, 10 mM glucose (pH 7.4) and incubated with Fluo 4-AM (500 µg/mL; Invitrogen) and Pluronic F-127 (0.1%; Invitrogen) at 37 °C for 45 min. Calcium imaging was performed for 30 min using a Multi-photon Zeiss LSM 7MP OPO microscope (Zeiss, Marly le Roi, France) and analyzed with the ZEN software (Zeiss).

Calcineurin activity was measured using the calcineurin cellular activity assay kit (Enzo, Villeurbane, France). Day 3-micropellets were rinsed in Tris Buffered Saline (TBS), lysed in the lysis buffer containing the protease inhibitor cocktail and grinded using an Ultra-turrax dispenser. Samples were then stored at −80 °C before quantification of calcineurin cellular activity by measuring the Ca^2+^/calmodulin-dependent Ser/Thr protein phosphatase 2B (PP2B) activity according to manufacturer’s instructions.

### Statistical analysis

Statistical analysis was performed using GraphPad Prism 7 software (San Diego, USA). Data were represented as mean ± standard error of the mean (sem) of separate experiments. Data did not assume a Gaussian distribution and were analyzed using appropriate nonparametric statistical tests. The comparison between 2 unpaired groups was performed using a Mann–Whitney test. The comparison between one group compared to the control group normalized to 1 or 100% was performed using a Wilcoxon signed rank test. The comparison between different groups was analyzed by a Kruskall-Wallis test followed by a Dunn’s multiple comparisons test. Correlations were analyzed with a nonparametric Spearman test. Statistical differences were indicated as *: p < 0.05, **: p < 0.01, ***: p < 0.001.

## Supplementary Information

**Additional file 1: Fig. S1.** Role of NMB in chondrocytes. **A)** Fold change expression of *NMB* and chondrocyte markers in OA chondrocytes transfected with siCT or siNMB at day 3 of chondrogenesis. Results are expressed as mean ± sem (n = 3 biological replicates). **B)** Fold change expression of *NMB* and chondrocyte markers in OA chondrocytes cultured with the NMBR antagonist BIM23042 (50 nM) at day 3 of chondrogenesis. Results are expressed as mean ± sem (n = 6 biological replicates). *: p ≤ 0.05.

## Data Availability

Gene expression data from this study have been deposited at Gene Expression Omnibus.

## Abbreviations

ACAN: Aggrecan
ALPL: Alkaline phosphatase
BGLAP: Bone gamma-carboxyglutamate protein (osteocalcin)
BIM: BIM23042
BM-MSC: Bone marrow-derived MSC
CDKN2D: Cyclin-dependent kinase 4 inhibitor 2D
COL: Collagen
DAG: 1,2-Diacylglycerol
DMM: Destabilization of medial meniscus
ER: Endoplasmic reticulum
ETS2: ETS Proto-Oncogene 2
FABP: Fatty acid binding protein
FALPL: Lipoprotein lipase
GAG: Glycosaminoglycan
FKBP: FKBP Prolyl Isomerase
HEYL: Hes Related Family BHLH Transcription Factor with YRPW Motif Like
iP_3_: Inositol 1,4,5-triphosphate
ITPR1: Inositol 1,4,5-Trisphosphate Receptor Type 1
ITG: Integrin
JARID2: Jumonji and AT-Rich Interaction Domain Containing 2
KCNE4: Potassium Voltage-Gated Channel Subfamily E Regulatory Subunit 4
KCNH1: Potassium Voltage-Gated Channel Subfamily H Member 1
MSC: Mesenchymal stromal cell
NDN: Necdin
NFAT: Nuclear factor of activated T-cells
NFKBIA: NFKB Inhibitor Alpha
NMB: Neuromedin
NMBR: NMB receptor
NR4A1: Nuclear Receptor Subfamily 4 Group A Member 1
NRG: Neuregulin
OA: osteoarthritis
PDK: Pyruvate Dehydrogenase Kinase
PFKFB: 6-Phosphofructo-2-Kinase/Fructose-2,6-Biphosphatase
PIP_2_: Phosphatidyl inositol 4,5-biphosphate
PLAUR: Plasminogen Activator, Urokinase Receptor
PLC_β_: Isoform β of phospholipase C
PPAR: Peroxisome proliferation-activated receptor
PRRG4: Proline Rich and Gla Domain
RASD2: RASD Family Member 2
RGCC: Regulator of Cell Cycle
rNMB: Recombinant NMB
RUNX2: Runt-related transcription factor
siCT: SiRNA-control
siNMB: SiRNA specific for NMB
SLC19A2: Solute Carrier Family 19 Member 2
SOCE: Store-operated Ca^2+^ entry
SOX: SRY-box transcription factor
SPHK: Sphingosine Kinase
TGF: Transforming growth factor
TGM2: Transglutaminase 2
TIPARP: TCDD Inducible Poly(ADP-Ribose) Polymerase
TNFAIP: TNF Alpha Induced Protein
TRPV: Transient receptor potential vanilloid
TSPAN: Tetraspanin
VGCC: Voltage gated Ca^2+^ channels
ZFP: Zing finger protein
