# Supplementary figures and images for: Neuromedin B promotes chondrocyte differentiation of mesenchymal stromal cells via calcineurin and calcium signaling
Source: Cell Biosci. 2021 Oct 18;11:183. doi: 10.1186/s13578-021-00695-1 (PMC8525028; doi:10.1186/s13578-021-00695-1)

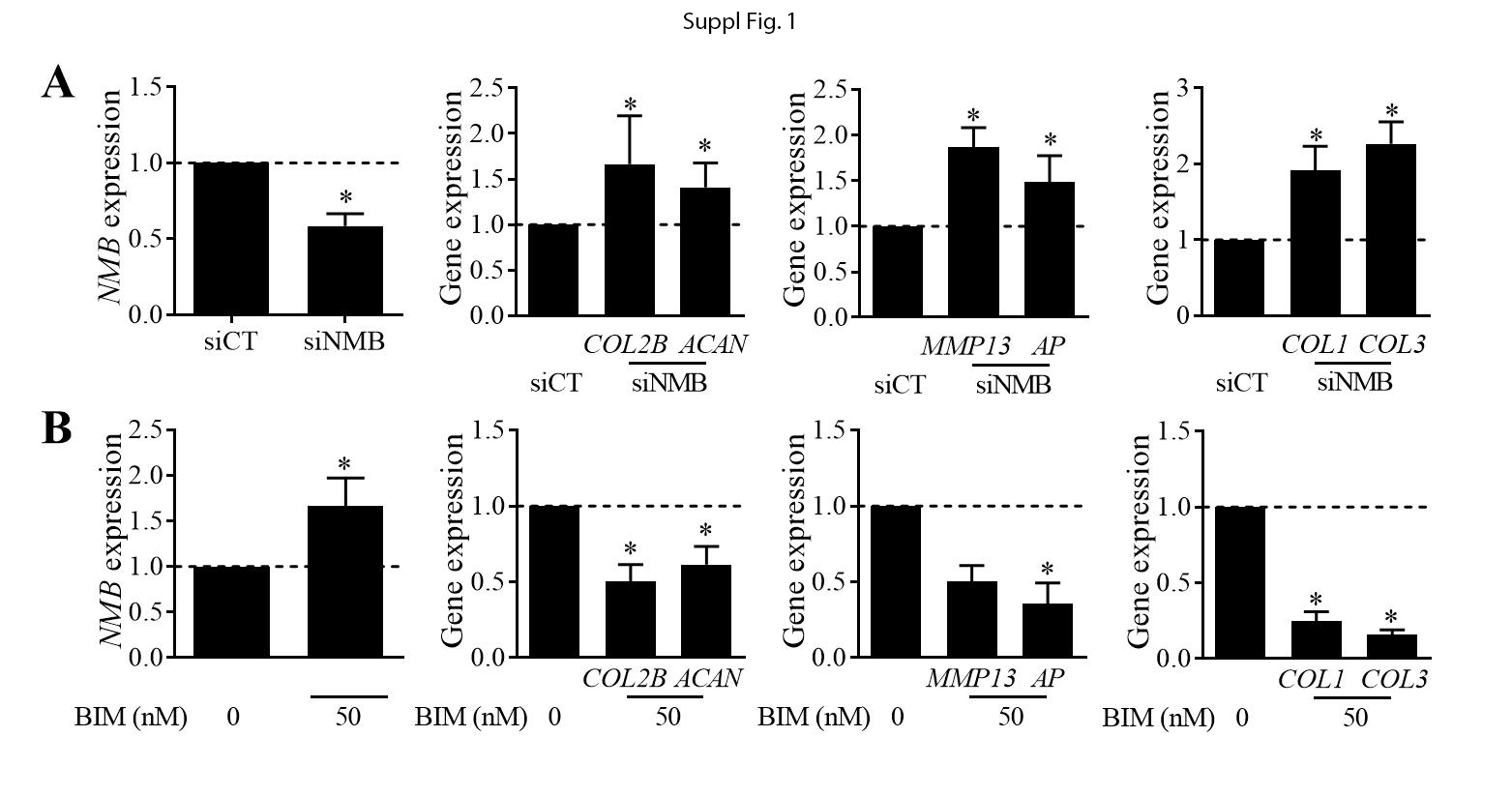

Supplement: Supplementary file 1 — Additional file 1: Fig. S1. Role of NMB in chondrocytes. A) Fold change expression of NMB and chondrocyte markers in OA chondrocytes transfected with siCT or siNMB at day 3 of chondrogenesis. Results are expressed as mean ± sem (n = 3 biological replicates). B) Fold change expression of NMB and chondrocyte markers in OA chondrocytes cultured with the NMBR antagonist BIM23042 (50 nM) at day 3 of chondrogenesis. Results are expressed as mean ± sem (n = 6 biological replicates). *: p ≤ 0.05. [file 13578_2021_695_MOESM1_ESM.jpg]
